# Searching on the Back: Attentional Selectivity in the Periphery of the Tactile Field

**DOI:** 10.3389/fpsyg.2022.934573

**Published:** 2022-07-13

**Authors:** Elena Gherri, Felicity White, Elisabetta Ambron

**Affiliations:** ^1^Dipartimento di Filosofia e Comunicazione, University of Bologna, Bologna, Italy; ^2^Human Cognitive Neuroscience, University of Edinburgh, Edinburgh, United Kingdom; ^3^Laboratory for Cognition and Neural Stimulation, Neurology Department, School of Medicine University of Pennsylvania, Philadelphia, PA, United States

**Keywords:** touch, selective attention, event-related potentials (ERP), N140cc, tactile search

## Abstract

Recent evidence has identified the N140cc lateralized component of event-related potentials as a reliable index of the deployment of attention to task-relevant items in touch. However, existing ERP studies have presented the tactile search array to participants' limbs, most often to the hands. Here, we investigated distractor interference effects when the tactile search array was presented to a portion of the body that is less lateralized and peripheral compared to the hands. Participants were asked to localize a tactile target presented among distractors in a circular arrangement to their back. The N140cc was elicited contralateral to the target when the singleton distractor was absent. Its amplitude was reduced when the singleton distractor was present and contralateral to the target, suggesting that attention was directed at least in part to the distractor when the singletons are on opposite sides. However, similar N140cc were observed when the singleton distractor was ipsilateral to the target compared to distractor absent trials. We suggest that when target and singleton distractor are ipsilateral, the exact localization of the target requires the attentional processing of all items on the same side of the array, similar to distractor absent trials. Together, these observations replicate the distractor interference effects previously observed for the hands, suggesting that analogous mechanisms guide attentional selectivity across different body parts.

## Introduction

To efficiently deal with complex sensory environments, our brain can engage selective attention mechanisms to prioritize the processing of relevant stimuli at the expense of irrelevant ones. In a typical tactile search task, simultaneous tactile stimuli are presented to the body and participants have to report the presence and/or process the features of the task-relevant tactile target while ignoring all tactile distractors (e.g., Overvliet et al., 2007; Toet et al., 2008; Assumpção et al., 2018; Halfen et al., 2020). Initial behavioral evidence revealed that search time increased as a function of the number of tactile distractors in the array (e.g., Toet et al., 2008; Halfen et al., 2020), suggesting that attention is moved serially from one item of the array to another until the target is identified.

However, the underlying neural mechanisms responsible for the selection of the task-relevant information in touch have been scarcely investigated and remain poorly understood. Electrophysiological studies have recently identified a lateralized ERP component, the N140cc (central contralateral), that appears to index the deployment of attention during tactile search tasks (e.g., Katus et al., 2015; Forster et al., 2016; Ambron et al., 2018; Katus and Eimer, 2019). The N140cc was first observed in a task requiring to detect the presence or absence of a target among homogenous distractors (stimuli differed with respect to their frequencies; Forster et al., 2016). The results revealed an enhanced negativity elicited over the hemisphere contralateral to the target side over central electrodes (close to somatosensory areas) starting from about 100 ms from the onset of the search array (Katus et al., 2015; Forster et al., 2016).

This finding was particularly intriguing because of the clear parallels with the well-established N2pc component observed during visual search tasks and considered the electrophysiological correlates of covert attentional deployment in vision (Luck, 2012; Woodman, 2013; Eimer, 2014). Typically, the presentation of the target within the visual search array elicits an increased negativity over occipital electrodes contralateral to the target as compared to ipsilateral ones from about 200–300 ms post-array onset (e.g., Luck and Hillyard, 1990, 1994; Eimer, 1996). The N2pc has been extensively used to investigate the experimental conditions under which selective attention is directed to relevant items in visual search tasks. For example, a salient but irrelevant distractor (singleton distractor) can attract attention, as indexed by the N2pc, interfering with target selection (Gaspar and McDonald, 2014; Gaspelin and Luck, 2018). Furthermore, when both the target and the singleton distractor are on the same side of the search array, the distractor interference increases as the target-distractor distance decreases (being maximal when they are next to each other) as shown by reduced N2pc amplitudes and increased RTs and error rates (e.g., Hilimire et al., 2009; Hilimire and Corballis, 2014). This suggests degraded target selection processes due to the competitive interactions between the target and the ipsilateral singleton distractor (Hilimire et al., 2009; Hilimire and Corballis, 2014).

Whether and to what extent visual and tactile attention mechanisms are functionally equivalent remains unclear. The effect of distractor interference in touch was recently investigated in an ERP study in which the tactile search array was delivered to two fingers of the left and right hand (Mena et al., 2020). The presence of an irrelevant singleton distractor delayed responses to the target (Mena et al., 2020). However, no difference emerged between the N140cc amplitudes elicited on *distractor absent trials (*target was presented with homogenous distractors) and on *ipsilateral distractor trials (*target ipsilateral to the singleton distractor; Mena et al., 2020). That is, there was no electrophysiological evidence for a degraded attentional selection of the target when target and singleton distractor were adjacent.

The effect of target-singleton distractor separation in touch was investigated in a separate study in which the distance between these singletons was manipulated within the same side of the tactile search array (Gherri et al., 2021). Target and singleton distractor were presented to contiguous or non-contiguous fingers of the same hand within a six-item search array in which three stimuli were delivered to the fingers of the right and of the left hand. In contrast to visual search studies, performance worsened and the N140cc amplitude increased when the singletons were delivered to non-contiguous as compared to contiguous fingers, suggesting additional attentional resources (or shifts within the same hand) for increased target-distractor separation. Thus, relevant information appears to compete for representation differently in vision and touch.

**Figure 1:**
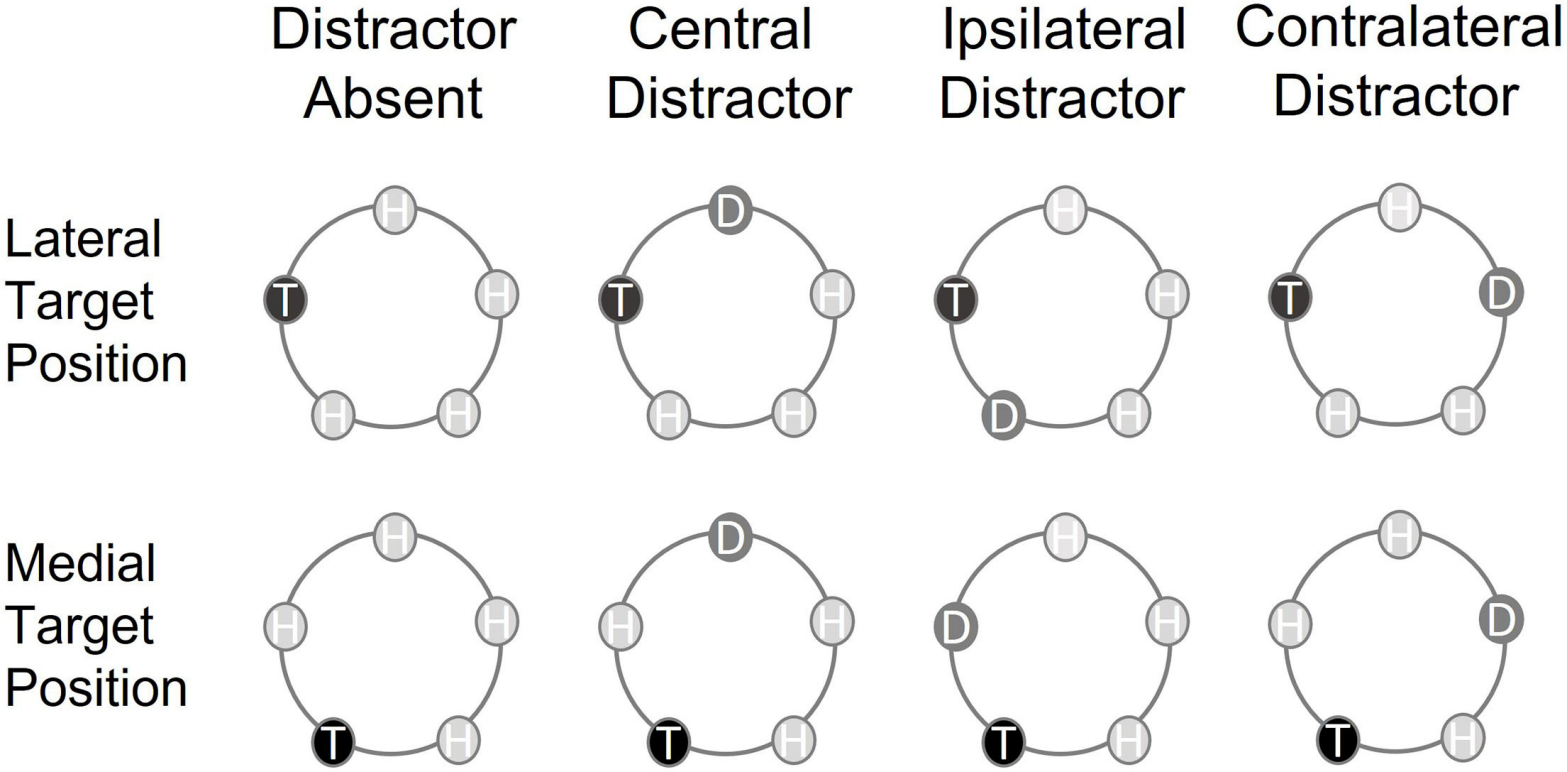
Schematic representation of the circular array and the target positions in the different types of trials included in the analyses. The tactile search array included one singleton target (T) and four homogeneous distractors (H) (*distractor absent trials*) or one singleton target (T), three homogeneous distractors (H), and one singleton distractor (D) (*distractor present trials*). The vertical labels refer to the presence and position within the array of the singleton distractor (type of trial), whereas the horizontal labels refer to the position of the target within the search array (target position).

Importantly, the attentional mechanisms deployed to select the tactile target may depend on the stimulated body location (Forster et al., 2016). So far, studies investigating the N140cc component have presented the tactile search arrays to distal body parts (e.g., Ambron et al., 2018; Katus and Eimer, 2019; Mena et al., 2020; Gherri et al., 2021), which are characterized by a higher density of mechanoreceptors in the skin and by smaller receptive fields (RFs) (e.g., Johansson and Vallbo, 1979). This study investigated the selection of task-relevant tactile information delivered to a proximal body part such as the participants' backs. First, we wanted to ascertain whether similar distractor-interference effects can be observed over the back where the larger size of the somatosensory RFs (Conti et al., 1986) increases the likelihood of “ambiguous” neuronal responses (e.g., Luck et al., 1997) elicited by multiple tactile stimuli falling within the same RFs. We asked participants to localize a tactile target presented within a five-item search array and investigated whether the N140cc elicited by target selection was modulated by the presence and position of a singleton distractor. Tactile stimuli were defined by different frequencies (target 100 Hz, singleton distractor 20 Hz, and homogeneous distractors 10 Hz) and were arranged in a circular shape, so that they were equidistant from a central point located on the midline of participants' back (refer to Figure 1). The comparison between trials in which the target was surrounded by homogeneous distractors with those in which the target was ipsilateral or contralateral to the singleton distractor will offer new insights into whether the attentional mechanisms described for the hands (Mena et al., 2020) can also account for tactile selectivity on the back.

Second, presenting the search array to the back allowed us to explore questions concerning the anchor of tactile selective attention and its movements within and across the different body sides. Studies on *tactile spatial attention* have shown that attention to locations operates upon a multisensory representation of space which is modulated by the position of the eyes, head, and stimulated limbs in external space (e.g., Eimer et al., 2004; Gillmeister et al., 2010; Heed and Röder, 2010; Gillmeister and Forster, 2012; Gherri and Forster, 2014). Tactile spatial attention has been suggested to operate upon an action-based reference frame anchored to the eyes for tactile targets delivered to visible body locations and to the head or trunk for targets presented to non-visible parts of the body (such as the back) (Heed and Röder, 2011). However, non-visible tactile targets were also found to be represented according to an external (proprioceptive) reference frame, anchored to the center of visible space and “wrapped” around the body, extending along the dorsoventral axis from the front body midline to the center of the back (Gillmeister and Forster, 2012).

It remains unknown whether analogous attentional anchors are employed to guide *selective attention* in touch. In this study, the search array presented to non-visible space on the back allowed us to test between two alternative possibilities. Because all tactile stimuli were equidistant from a central point on the midline of participants' back, no difference should emerge between different target locations if attention is initially allocated to the center of the search array on the back. However, in our search array, two of the stimuli were closer to the back midline (“medial” positions) whereas two “lateral” stimuli were further away from it (refer to Figure 1; one stimulus was directly on the midline). If selective attention is initially anchored to visible space in the front and moves from the front to the back of the body along external space, systematic differences should emerge between lateral and medial targets due to their different eccentricities relative to the anchor of attention.

## Method

### Participants

A total of 23 paid, healthy volunteers took part in the study. Three participants were excluded because of low accuracy. Hence, 20 participants remained in the sample (11 females, aged 20–36 years, average age: 24.5 years). All but three were right-handed by self-report. The experiment was approved by the PPLS Research Ethics Committee, Department of Psychology, Edinburgh.

### Stimuli and Procedure

Participants were tested in a dimly lit sound-attenuated chamber. A total of five tactile stimulators were attached to participant's back and arranged in a circular array (Figure 1) in which each stimulator was equidistant from the center. The uppermost stimulator was placed at the body midline, just below the level of the first thoracic vertebra. A total of two stimulators were placed level with the middle of the shoulder blade, and two levels with the middle back, in such a way that all tactile stimulators were equidistant from one another (14 cm apart). A total of 12 V solenoids were used to produce tactile stimuli. Whenever an electric current passed through the solenoids, a plastic rod with a conical tip was pressed lightly against the skin.

The search array consisted of the simultaneous presentation of five tactile stimuli: a “target,” a “singleton distractor” and three “homogenous distractors.” The target frequency (100 Hz) was generated by switching the solenoid ON for 5 ms and OFF for 5 ms for 80 cycles. For the singleton distractor frequency (20 Hz), the solenoid was ON for 5 ms and OFF for 45 ms for 8 cycles, whereas for the homogeneous distractor frequency (10 Hz), the solenoid was ON for 5 ms and OFF for 95 ms for 4 cycles. The duration of the search array was 405 ms. Each trial started with the onset of the search array and was followed by a 1,500 ms empty interval in which responses were recorded.

The tactile search array either included one target and four homogenous distractors (*distractor absent trials*) or one target, one singleton distractor, and three homogenous distractors (*distractor present trials*). On *distractor absent* trials, the target was presented at one of the five possible locations, whereas the homogenous distractors were presented at all other locations. On *distractor present* trials, the target was presented at one location, the singleton distractor at a second location, and the homogenous distractors at the remaining three locations. Thus, there were four different types of distractor present trials: lateralized target and central singleton distractor on the midline (*central distractor*), lateralized target and singleton distractor on the same side of the midline (*ipsilateral distractor*), lateralized target and singleton distractor on the opposite side of the midline (*contralateral distractor*), and central target on the midline and lateralized singleton distractor (*lateralized distractor*).

Each block contained 84 trials in total, 20 distractor absent trials (4 repetitions for each of the five possible target locations), and 64 distractor present trials (4 repetitions for each of the four possible distractor present trial types). The target and the singleton distractor were presented with equal probability in each of the array locations. All responses were equally likely. Trials were presented in a random order within each block, with 10 blocks in total (840 trials).

Trials in which the target was presented on the midline were excluded from all analyses because no target-elicited lateralized component can be elicited by stimuli over the midline. In addition, trials with a central target and a lateralized singleton distractor (*lateralized distractor trials*) were also excluded because preliminary analyses revealed that no significant ERP lateralizations were elicited by the singleton distractor on these trials (*p* > 0.34).

Participants' task was to identify the exact position of the target within the search array and press the corresponding location on a five-button finger push pad. Left to right on the push pad corresponded to left to right on the back, going clockwise from lower left tactile stimulator round to lower right tactile stimulator. Participants were instructed to keep all five fingers on the pad during the block to ensure consistency in motor response. The hand used to respond was counterbalanced within participants, alternating left and right between blocks.

During the experimental blocks, participants kept fixation on a central point aligned with their body midline placed 60 cm in front of them. Compliance was monitored by the experimenter *via* a video camera. White noise at 65 dB was presented throughout the experimental blocks to mask any sound made by the tactile stimulators. Practice trials consisted in four repetitions of each of the stimuli frequencies delivered sequentially in each location of the search array, followed by a training block of 42 trials after which participants received performance feedback.

### EEG Recording

Electroencephalography (EEG) was recorded using a BIOSEMI ActiveTwo amplifier system from 64 active electrodes positioned according to the 10–20 system. A total of two additional electrodes were placed on the earlobes. Horizontal eye movements (HEOGs) were recorded from two electrodes placed at the outer canthi of each eye. Vertical eye movements (VEOGs) were recorded from two electrodes positioned above and below the right eye. The EEG was sampled at 512 Hz.

Electroencephalography data were analyzed using Brain Vision Analyser (version 2.0.4.368). HEOG and VEOG were computed offline as bipolar channels. EEG was digitally re-referenced to the average of the left and right earlobe and was digitally filtered offline (high-pass filter 0.53 Hz, low-pass filter 40 Hz, and notch filter 50 Hz). The EEG was epoched into 500-ms intervals starting 100 ms before and ending 400 ms after the search array onset. Trials with eye blinks (voltage exceeding ±60 μV on the VEOG channel), horizontal eye movements (voltage exceeding ±40 μV on the HEOG channel), and other artifacts (voltage exceeding ±80 μV at all other electrode sites) were excluded from further analysis.

**Figure 2:**
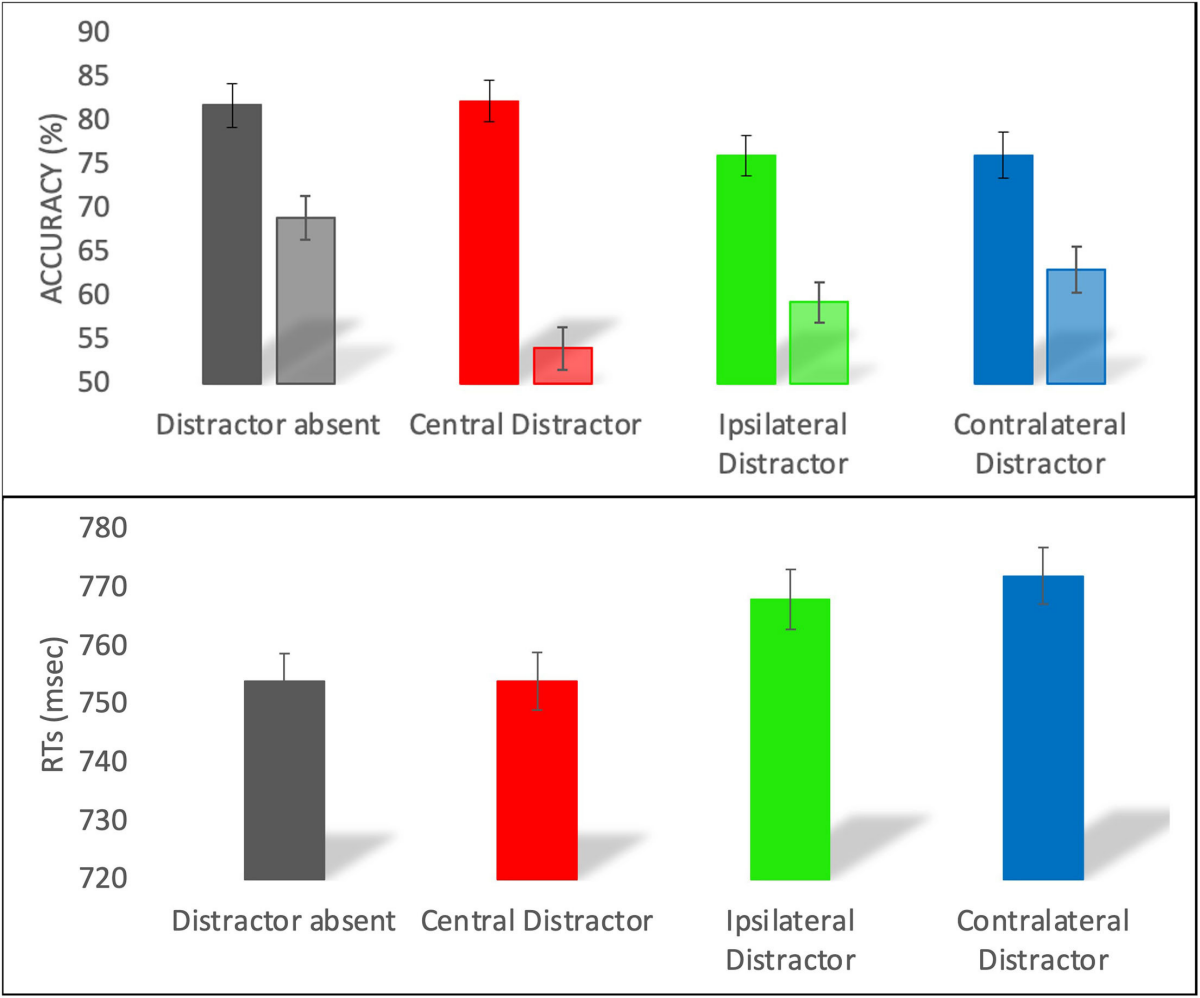
Accuracy (top panel) and RT (bottom panel) in the distractor absent, central distractor, ipsilateral distractor, and contralateral distractor trials. For the accuracy, the darker colors represent the lateral targets and the lighter colors represent the medial targets. Error bars represent the standard errors of the means.

Event-Related Potentials (ERPs) were averaged relative to a 100 ms pre-stimulus baseline separately for all combinations of types of trial (distractor absent vs. central distractor vs. ipsilateral distractor vs. contralateral distractor) and target position (medial vs. lateral position within the search array). ERP mean amplitude values were computed for each participant at electrodes C5/6 (where the lateralized components of interest were maximal in this study, refer to Figure 2), within two successive measurement windows 110–240 and 250–400 ms post-stimulus onset (Forster et al., 2016; Ambron et al., 2018).

### Data Analyses

Accuracy and reaction times (RTs) data were analyzed using linear mixed model (LMM) in R (3.3.0). Factors of interest were type of trial (distractor absent vs. central distractor vs. ipsilateral distractor, vs. contralateral distractor) and target position (the position of the target with respect to the midline, lateral vs. medial). Independently run LMM analyses tested the contribution of the fixed factor(s) of interest to the model fit. To do so, we compared a baseline model with only subjects as random intercepts with the same model including the factor(s) of interest using the ANOVA function. Factors of interest were inserted in a stepwise manner. In the Result section, we only reported the final models including the factors that contributed significantly to the model fit, as compared to the model including only one significant factor of interest.

For the ERP data, repeated-measures analyses of variance (ANOVA) were conducted for the factor laterality (hemisphere contralateral vs. ipsilateral to the target side), type of trial (distractor absent vs. central distractor vs. ipsilateral distractor vs. contralateral distractor), and target position (lateral vs. medial). In these analyses, the presence of reliable lateralized components is reflected by the main effect of the factor laterality, indicating the significant differences between the hemisphere contralateral and ipsilateral to the target side. Following significant laterality x type of trial interactions, separate analyses were first carried out for each type of trial, to determine the presence of significant N140cc lateralized components. The amplitude of these N140cc was then calculated by subtracting the ERPs elicited at electrodes ipsilateral to the target from contralateral ERPs, separately for the different types of trials. Finally, we run planned contrasts between the different types of trials to determine whether the target-elicited N140cc amplitude was modulated by the presence and location of the singleton distractor. Likewise, following significant laterality x target position interactions, separate analyses were carried out for each target position (lateral vs. medial), to determine the presence of significant N140cc lateralized components.

## Results

### Behavioral Results

Mixed linear model showed that both target position and type of trial contributed significantly to the model fit of both accuracy and RT.

For the accuracy, the model with interaction between target position and type of trial was a better predictor than the main effects only model (logLik = −7036, χ^2^ (3) = 58, *p* < 0.001). Participants were overall more accurate with the lateral (*M* = 79, *SE* = 2.3) than medial (*M* = 61, *SE* = 4.4) target. As shown in Figure 2 (top panel), when the target was in a medial position, we observed a significant difference across all types of trials (*z* > 3, *p* < 0.005), with distractor absent more accurate than contralateral, which was in turn more accurate than ipsilateral and central distractor trials. For lateral targets, we observed a significant difference across all conditions (*z* > 3, *p* < 0.005), except for the comparisons between distractor absent and central distractor (*z* = 0.33, *p* = 0.74), and between contralateral and ipsilateral distractor (*z* = 0, *p* = 1).

For the RTs, the final model included only the main effects of target position and type of trial, as the model including the interaction did not improve the model fit (logLik = −57256, χ^2^ (3) = 2.8, *p* = 0.41). RTs were faster for lateral (*M* = 752, *SE* = 3.2) as compared to medial (*M* = 775, *SE* = 3.7) targets. RTs were faster on distractor absent trials than on ipsilateral (*t* = 2.6, *p* = 0.008) and contralateral (*t* = 3.4, *p* < 0.001) distractor trials, whereas similar RTs were observed on distractor absent and central distractor trials (*t* = 0.8, *p* = 0.38) and on contralateral and ipsilateral distractor trials (*t* = 0.7, *p* = 0.48). Central distractor trials differed only from contralateral distractor ones (*t* = 2.4, *p* = 0.01).

### ERP Results

**Figure 3:**
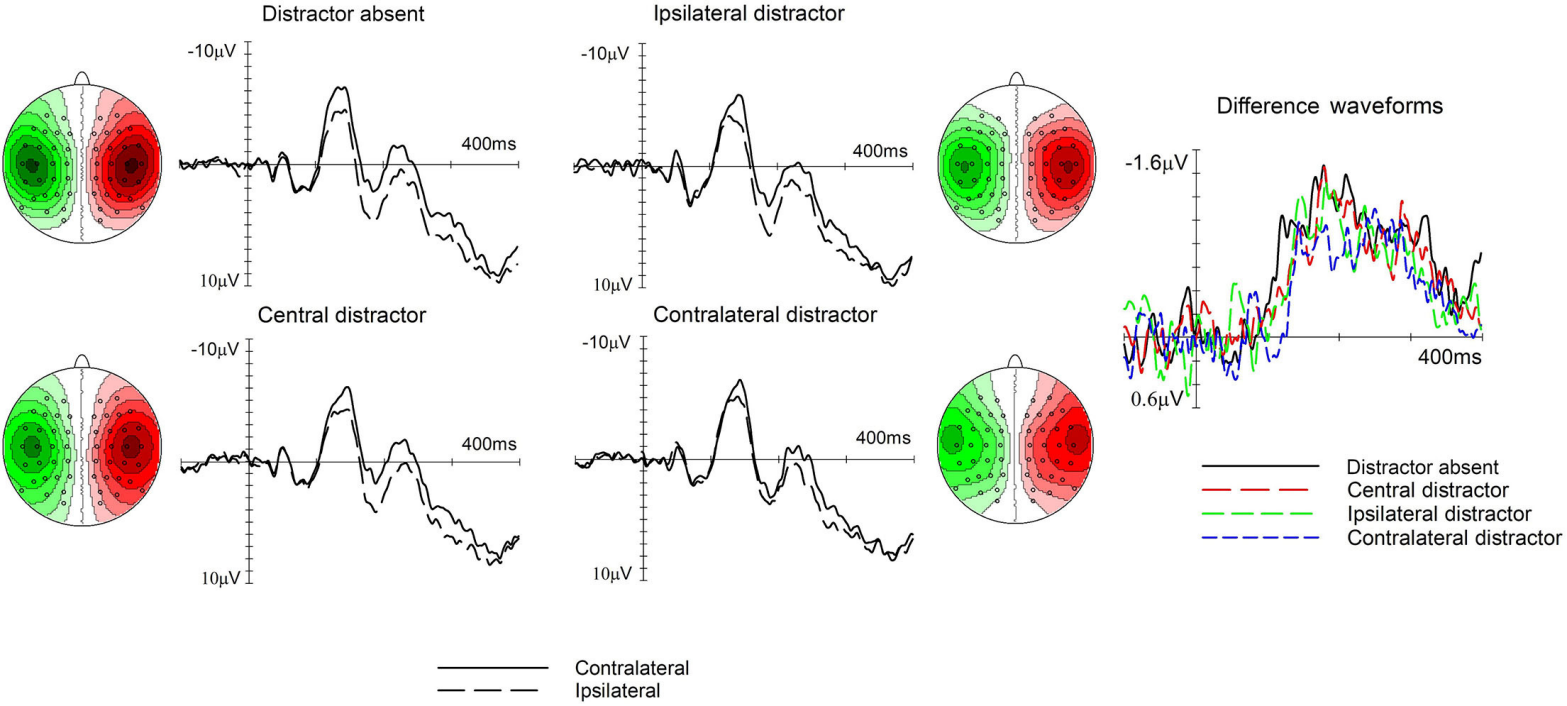
The effect of trial type on the N140cc lateralized component. Left and middle panels show ERPs elicited over electrodes C5/6 contralateral (solid line) and ipsilateral (dashed line) to the target side separately for the distractor absent, central distractor, ipsilateral distractor, and contralateral distractor trials. The corresponding difference waveforms are shown in the right panel. The N140cc scalp distribution is depicted separately for each type of trial. These are shown for the 110–240-ms interval and the values range between ± 1.2 μV.

**Figure 4:**
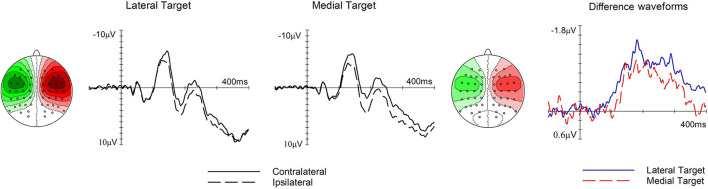
The effect of target position on the N140cc lateralized component. Left and middle panels show ERPs elicited over electrodes C5/6 contralateral (solid line) and ipsilateral (dashed line) to the target side when the target was presented at lateral and medial positions, respectively. The corresponding difference waveforms are shown in the panel on the right. The N140cc scalp distribution is shown separately for each target position. These relate to the 250–400-ms time window and the values range between ± 1.1 μV.

Figures 3, 4, left and middle panels, show somatosensory ERP waveforms elicited by the tactile search array over the hemisphere contralateral and ipsilateral to the target side at electrode pair C5/6, whereas the corresponding difference waveforms are shown in the right panel. Figure 3 shows the effect of type of trial on the N140cc amplitude, whereas Figure 4 shows the effect of target position. Both figures also show the N140cc scalp distributions.

In the 110–240-ms time window, a main effect of laterality (*F*_(1, 19)_ = 76.2, *p* < 0.001, ηp^2^ = 0.8) reflected the presence of reliable N140cc components. Furthermore, the interaction between laterality and type of trial was significant (*F*_(2.6, 50.2)_ = 3, *p* = 0.043, ηp^2^ = 0.138), refer to Figure 3). First, we checked for the presence of statistically significant N140cc components by comparing the mean amplitude values for ERPs elicited over contralateral and ipsilateral electrodes, separately for the different types of trials (all *t*_(19)_ > 5.1, all *p* < 0.001). Then, to investigate the differences between the lateralized components observed on different trials, the mean amplitude of the N140cc component was calculated separately for each trial type. Bonferroni corrected pairwise contrasts were then carried out on these values. Larger N140cc amplitudes were observed on distractor absent trials (M = −1.08, SD = 0.64) as compared to contralateral distractor trials (M = −0.6, SD = 0.54; *t*_(19)_ = −3.1), *p* = 0.006). Finally, no laterality x target position (*F*_(1, 19)_ = 2.1, *p* = 0.15, ηp^2^ = 0.1) nor laterality x trial type x target position (*F*_(2.7, 52.7)_ = 0.5, p = 0.6, ηp^2^ = 0.028) interactions were present in this time window.

In the following time window (250–400 ms), the presence of the later phase of the N140cc was substantiated by a significant main effect of laterality (*F*_(1, 19)_ = 32.6, *p* <0.001, ηp^2^ = 0.63). No laterality x type of trial interaction was present (*F*_(2.5, 46.7)_ = 0.7, *p* = 0.6, ηp^2^ = 0.035). However, a significant laterality x target position (*F*_(1, 19)_ = 10.08, p <0.005, ηp^2^ = 0.35) revealed a larger N140cc when the target was in a lateral (M = −0.74, SD = 0.56) as compared to a medial position (M = −0.37, SD = 0.43), refer to Figure 4. The N140cc was significantly present for lateral targets (*t*_(19)_ = 3.7, *p* = 0.001) and medial targets (*t*_(19)_ = 5.8, *p* < 0.001). No three-way interaction was present in this time window (*F*_(2.27, 43.3)_ = 0.56, *p* = 0.59, ηp^2^ = 0.029).

## Discussion

In this study, we investigated the attentional mechanisms responsible for target selection, as indexed by the N140cc lateralized component, in a tactile search task in which a five-items search array was presented to the participants' back. The presence of reliable N140cc components contralateral to the target side on distractor absent trials (i.e., target surrounded by homogeneous distractors) suggests that analogous mechanisms are engaged to select task-relevant information presented to the hands/limbs as to a “perceptually peripheral” part of the body. Importantly, the presence of a *singleton distractor* in a lateralized position of the search array (i.e., to the left or right of the body midline, that is on ipsilateral or contralateral distractor trials) affected target selection and modulated the target-elicited N140cc component. Responses were slower and less accurate on ipsilateral and contralateral distractor trials compared to distractor absent trials, suggesting that the presence of the singleton distractor in a lateralized position delayed the localization of the target. Furthermore, a reduced N140cc amplitude observed on contralateral distractor compared to distractor absent trials likely reflects the allocation of part of the attentional resources to the side of the singleton distractor which was opposite to the target side. The results also revealed the absence of reliable differences between the N140cc amplitudes on distractor absent and ipsilateral distractor trials, suggesting a similar allocation of attentional resources to the target side when the lateralized target was next to a homogenous distractor (on distractor absent trials), or to a singleton distractor (on ipsilateral distractor trials).

Central distractor trials are interesting from a methodological perspective because singletons presented over the midline do not trigger lateralized ERP components (e.g., Woodman and Luck, 2003), allowing the isolation of the lateralized components elicited by the lateralized target in the presence of a central singleton distractor. The localization response speed of the lateralized target was not delayed by the presence of the central singleton distractor, and similar N140cc amplitudes were elicited on central distractor and on distractor absent trials. Thus, the singleton distractor was easier to ignore when presented over the midline compared to the other lateralized locations on the back (on ipsilateral and contralateral distractors trials), suggesting that the singleton distractor over the midline can be quickly discounted as irrelevant (it is processed similarly to the homogeneous distractors).

Based on the present results, we speculate that participants pre-attentively identify perceptual discontinuity between the two sides of the tactile array. This discontinuity indicates the likely side of the target and is used to inform the allocation of attention to one side, as indexed by the N140cc, for in-depth stimulus processing. The exact localization of the target may require an attentional analysis of all items on the same side, resulting in similar N140cc amplitudes when attention is initially directed to the correct target side. The results of this study corroborate and expand the initial results observed for the tactile search on the hand (Mena et al., 2020), suggesting that similar mechanisms also operate in the periphery of the tactile field (i.e., the back). However, differences between distractor present and distractor absent trials were less pronounced in this study. One possible explanation is that the attentional mechanisms engaged to select the tactile target on the back are less sensitive than those engaged on the hands. However, to keep performance well above the chance level, the difference between the frequencies of the target (100 Hz) and the singleton distractor (20 Hz) was increased compared to the Mena et al.'s study (in which the singleton distractor had a frequency of 40 Hz). Therefore, it is possible that the present results were in part driven by the decreased saliency of the singleton distractor.

Results also revealed an interesting effect of target “eccentricity” for targets presented further away from the back midline (lateral targets) compared to those closer to it (medial targets). This difference suggests that tactile attention was not anchored to the center of the circular array on the back because all stimuli within the array were equidistant from this central position, and no difference should have emerged between lateral and medial targets. Instead, targets presented further away from the back midline were easier to localize and elicited a larger N140cc components as compared to targets presented closer to the back midline. In visual search tasks, attention is assumed to be anchored initially to the central fixation point and then to move laterally, occupying successive locations beginning with near fixation targets followed by far fixation targets, as revealed by N2pc studies (e.g., Woodman and Luck, 2003). The pattern of result observed in this study could suggest that attention was initially allocated to the frontal visible space (possibly anchored to gaze direction), moving along the dorsoventral axis from the front body midline to the center of the back (c.f. Gillmeister and Forster, 2012). Thus, lateral tactile targets were selected more easily than medial ones because they were closer to the anchor of attention. While intriguing, this hypothesis is highly speculative and needs to be further substantiated in the future studies. Thus far, the processing and localization of tactile stimuli delivered to the back remains poorly understood as highlighted by initial behavioral evidence which suggests that tactile stimuli presented to the back midline are not simply encoded as stimuli delivered to the center of the body (Van Erp, 2005; Haggard and Giovagnoli, 2011; Salzer et al., 2014).

The effect of target eccentricity was similar across all types of trials for both ERP and RT results, suggesting that it was primarily driven by the target location within the array. Interestingly, the difference between accuracy on medial and lateral targets was particularly evident on *central distractor trials* compared to other trial types. On these trials, performance was highly accurate for lateral targets (adjacent target and singleton distractor) but dropped significantly for medial targets (non-adjacent target and singleton distractor), improving when the target—singleton distractor separation decreased within the array. This observation replicates results observed when the target-distractor distance was selectively manipulated in the hand search task (Gherri et al., 2021). Thus, target localization improves when the singleton distractor is presented next to the target regardless of the stimulated body part.

One final consideration concerns the label used to describe the ERP lateralizations observed in the present and previous tactile search studies. We adopted the label “N140cc” to highlight that the onset of this lateralized component coincided with the somatosensory N140 ERP component (c.f. Forster et al., 2016), whereas other authors used the “N2cc” label to highlight the functional equivalence with the visual N2pc (e.g., Katus et al., 2015; Katus and Eimer, 2019). However, it is becoming apparent that this ERP lateralization is likely to include more than a single attentional process, not only because of its long duration but also because increasing evidence shows that the early and late phases of the N140cc are sensitive to different experimental manipulations. For these reasons, a broader label such as “central contralateral negativity” is likely to better reflect the functional correlates of this lateralized ERP component.

## Data Availability Statement

The raw data supporting the conclusions of this article will be made available by the authors, without undue reservation.

## Ethics Statement

The studies involving human participants were reviewed and approved by PPLS research Ethics Committee, University of Edinburgh. The patients/participants provided their written informed consent to participate in this study.

## Author Contributions

EG and EA ideated the study and analyzed the data. EG wrote the manuscript. EA revised it. FW programmed the experiment and collected the data. All authors contributed to the article and approved the submitted version.

## Funding

The work described in this manuscript was supported by a Carnegie Research Incentive Grant (RIG007519) and by a University of Edinburgh PPLS internal grant awarded to EG. Funding from the University of Bologna will cover the publishing fees.

## Conflict of Interest

The authors declare that the research was conducted in the absence of any commercial or financial relationships that could be construed as a potential conflict of interest.

## Publisher's Note

All claims expressed in this article are solely those of the authors and do not necessarily represent those of their affiliated organizations, or those of the publisher, the editors and the reviewers. Any product that may be evaluated in this article, or claim that may be made by its manufacturer, is not guaranteed or endorsed by the publisher.
